# The chitosan/carboxymethyl cellulose/montmorillonite scaffolds incorporated with epigallocatechin-3-gallate-loaded chitosan microspheres for promoting osteogenesis of human umbilical cord-derived mesenchymal stem cell

**DOI:** 10.1186/s40643-022-00513-7

**Published:** 2022-04-02

**Authors:** Jin Wang, Wubo He, Wen-Song Tan, Haibo Cai

**Affiliations:** State Key Laboratory of Bioreactor Engineering, East China University of Science and Technology, Shanghai, 200237 People’s Republic of China

**Keywords:** Epigallocatechin-3-gallate, Chitosan microspheres, Scaffolds, Osteogenesis

## Abstract

Epigallocatechin-3-gallate (EGCG) is a plant-derived flavonoid compound with the ability to promote the differentiation of human bone marrow-derived mesenchymal stem cells (MSCs) into osteoblasts. However, the effect of EGCG on the osteogenic differentiation of the human umbilical cord-derived mesenchymal stem cells (HUMSCs) is rarely studied. Therefore, in this study, the osteogenic effects of EGCG are studied in the HUMSCs by detecting cell proliferation, alkaline phosphatase (ALP) activity, calcium deposition and the expression of relevant osteogenic markers. The results showed that EGCG can promote the proliferation and osteogenic differentiation of the HUMSCs in vitro at a concentration of 2.5–5.0 μM. Unfortunately, the EGCG is easily metabolized by cells during cell culture, which reduces its bioavailability. Therefore, in this paper, EGCG-loaded microspheres (ECM) were prepared and embedded in chitosan/carboxymethyl cellulose/montmorillonite (CS/CMC/MMT) scaffolds to form CS/CMC/MMT-ECM scaffolds for improving the bioavailability of EGCG. The HUMSCs were cultured on CS/CMC/MMT-ECM scaffolds to induce osteogenic differentiation. The results showed that the CS/CMC/MMT-ECM scaffold continuously released EGCG for up to 22 days. In addition, CS/CMC/MMT-ECM scaffolds can promote osteoblast differentiation. Taken together, the present study suggested that entrainment of ECM into CS/CMC/MMT scaffolds was a prospective scheme for promotion osteogenic differentiation of the HUMSCs.

**Graphical Abstract:**

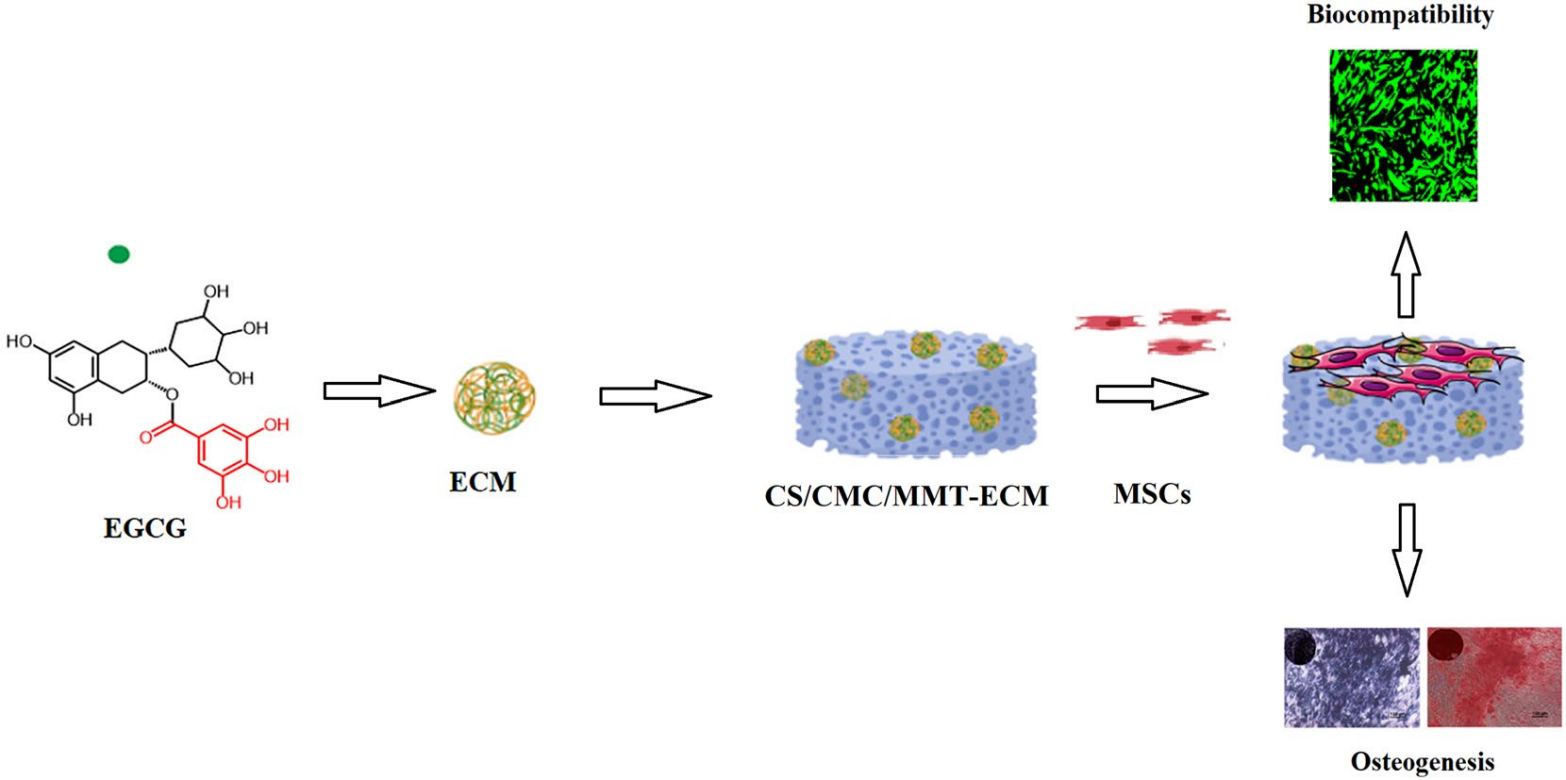

## Introduction

Mesenchymal stem cells (MSCs) are a type of adult stem cells originally reported to exist in the stroma of bone marrow (Ullah et al. 2015). In addition, MSCs have been isolated from many adult tissues, such as adipose tissue (Cabezas et al. 2018), synovial membrane (Neybecker et al. 2020), dental tissue (Hung et al. 2011), and umbilical cord (Zhang et al. 2020). MSCs exhibit distinctive stem cell properties of self-renewal and multi-lineage differentiation, and can differentiate into mesodermal lineage such as osteocytes, adipocytes chondrocytes, ectodermal neurocytes and endodermal lineages hepatocytes (Cheng et al. 2019). MSCs are also widely used in stem cell therapy and regenerative medicine due to their low immunogenicity, lack of ethical concerns, and immunoregulatory function (Gu et al. 2015; Rostami et al. 2020; Shariati et al. 2020). Recently, MSCs have also demonstrated the ability to repair bone tissue (Kong et al. 2019; Lei et al. 2012; Liu et al. 2020; Zhang et al. 2019).

The human umbilical cord-derived mesenchymal stem cells (HUMSCs) can differentiate into osteoblasts and have been used to repair bone defects (Kosinski et al. 2020; Yang et al. 2020a, b). The HUMSCs have certain advantages for clinical application. For instance, they are easily isolated; derived from the umbilical cord after birth, so the source of the cells is less controversial; replicate faster in vitro; the source of the cells is quite young; and less immune reactive after transplantation (Nagamura-Inoue et al. 2014). Several protocols have been established to direct the differentiation of the HUMSCs into osteoblasts, including the use of β-glycerophosphate, dexamethasone, and ascorbic acid (Fabian and Langenbach 2013; Freeman et al. 2016). However, the efficiency of osteogenic differentiation of MSCs in vitro still needs to be improved (Pittenger et al. 2019). Several bioactive molecules have been used to enhance the osteogenic differentiation of MSCs, such as growth factors (Safari et al. 2021; Su et al. 2014), cytokines (Hosogane et al. 2010), hormones (Lu et al. 2017), pharmaceuticals (Cui et al. 2017), phytochemicals compounds (Menon et al. 2018). Among them, phytochemicals compounds have attracted much attention because of their extensive sources and non-toxic side effects. Flavonoids are phytochemical compounds, which potentially promote the differentiation of MSCs into osteoblasts by activating signaling pathways associated with osteogenesis (Chen et al. 2018; Jin et al. 2014; Kulandaivelu et al. 2016; Zhou et al. 2015).

Epigallocatechin-3-gallate (EGCG) is a natural flavonoid found in green tea and has been reported to be involved in bone metabolism (Jin et al. 2014; Wang et al. 2016). Recent researches have confirmed that EGCG promotes proliferation and differentiation of human bone marrow-derived MSCs into osteoblasts (Wang et al. 2016). Further studies showed that EGCG promoted osteogenic differentiation through the activation of Wnt/β-catenin signaling pathway (Lee et al. 2013; Xi et al. 2018). However, the effect of EGCG on the osteogenic differentiation of HUMSCs is rarely studied. In addition, EGCG is easily affected by factors such as oxidant level, pH and temperature, and is rapidly metabolized by cells (Hou et al. 2005; Sato et al. 2017). Therefore, the sustained release of EGCG is of great significance in promoting the osteogenic differentiation of MSCs.

Drug-loaded microspheres are the preferred sustained-release system, because it can provide a large surface area to volume ratio, control the release time and improve the release effect (Yang et al. 2016). However, there are some obstacles that prevent microspheres from being directly used in the culture of HUMSCs, such as insufficient release time, small particle size that is not conducive to cell adhesion (Yang et al. 2020a, b). Natural polymer scaffolds are often used for three-dimensional (3D) culture of stem cells in vitro, which usually provides a more complete picture of cell-to-cell and cell–matrix interactions, better simulating the natural environment of the stem cells than traditional two dimension (2D) culture. In addition, many desirable cellular characteristics were maintained or even promoted in 3D culture (Vila-Parrondo et al. 2020; Wu et al. 2020; Ylostalo 2020). Therefore, the drug-loaded microspheres were wrapped into the scaffold to achieve the purpose of EGCG sustained release and provide adhesion carriers for cells.

Chitosan (CS) is biocompatible and linear cationic polymer (Li et al. 2016a, b). It is the partially deacetylated form of chitin, consisting of glucosamine and *N*-acetyl glucosamine with linkage (Komoto et al. 2019). In addition, CS is soluble under acidic conditions (Sun et al. 2019). They have also been reported to have antibacterial activity (Geisberger et al. 2013). Due to these advantageous properties, CS is widely used in the preparation of various carrier materials (Coimbra et al. 2011; Li et al. 2005). Carboxymethyl cellulose (CMC), which is very similar to CS in the structure and the cross-linking of CMC and CS plays an important role in improving the hydrophilicity, swelling and protein adsorption properties of CS (Menon et al. 2018b; Sainitya et al. 2015; Sun et al. 2019). CS/CMC scaffolds have also been used in cell cultures (Liu et al. 2009). Montmorillonite (MMT), the main component of bentonite, has been approved by the FDA as an additive in a variety of pharmaceutical products (Haroun et al. 2009; Katti et al. 2008). MMT has received significant attention in recent years due to favorable properties such as biocompatibility, availability and feasibility. In addition, Extensive research has shown that the introduction of MMT-prepared scaffolds into natural biomaterial (including gelatin, collagen, and chitosan) improves cell–scaffold interactions, cell proliferation, and cell differentiation (Hsu et al. 2012; Kevadiya et al. 2014; Nistor et al. 2015; Thakur et al. 2015).

In this study, the influence of EGCG on the HUMSCs in vitro culture was explored. Then EGCG was emulsified into chitosan microspheres. CS/CMC/MTT scaffolds were prepared by conventional freeze-drying approach. CS/CMC/MTT scaffolds were used as the matrix for loading EGCG-encapsulated chitosan microspheres (ECM). The scaffolds were characterized and then used to study its influence on the proliferation and osteoblast differentiation of the HUMSC.

## Materials and methods

### Materials

Chitosan (CS) and carboxymethyl cellulose (CMC) were obtained from Shanghai Macklin Biochemical Technology. Montmorillonite (MMT, K10) and the 3-(4,5-dimethylthiazol-2yl)-2,5-diphenyltetrazolium bromide (MTT) were purchased from Sigma-Aldrich (St. Louis, MO). Paraffin, span 80, glutaraldehyde and isopropanol were obtained from Aladdin Biochemical Technology, Shanghai, China. EGCG (purity ≥ 98%, high-performance liquid chromatography) was obtained from yuanye Bio-Technology, Shanghai, China. A Cell Counting Kit-8 (CCK8) was purchased from Dojindo China Co., Ltd. (Shanghai, China). BCIP/NBT alkaline phosphatase color development kit and enhanced BCA protein assay kit were obtained from Beyotime Biochemical Technology, Shanghai, China. The calcium colorimetric assay kit and alkaline phosphatase (ALP) kit were obtained from Jiancheng Biochemical Technology, Nanjing, China. All other cell culture products and reagents were purchased from GIBCO unless otherwise specified. All chemicals were of reagent grade and used without any further purification. Ultrapure water (18.2 MΩ, Millipore Co., USA) was used in all solutions and reagents throughout the experiment.

### Preparation of microspheres and scaffolds

The preparation of chitosan microspheres was based on the reported method slightly modified. In short, 400 mg CS was dissolved in 20 mL 1% (v/v) glacial acetic acid solution. The 45.86 mg EGCG was dissolved in 1 mL DMSO solution, which was prepared into 10 mM EGCG solution. Then 100 μL 10 mM EGCG solution were added drop-wise to the CS solution. The 2 mL Span 80 was added to 80 mL liquid paraffin at 50 ℃ under stirring 800 rpm to obtain continuous oil phase. The chitosan solution was slowly added to the oil phase, and stirred for 1.5 h (h) to form a stable oil/water system. After that, 2 mL 25% (v/v) glutaraldehyde was slowly added to the system, and stirred for 30 min (min) to cross-link chitosan. The microspheres suspension was centrifuged at 1500 rpm for 5 min to remove the supernatant. The microspheres were washed with isopropanol, ethanol and deionized water at room temperature. Finally, the ECM were obtained by freeze-drying. CS microsphere (CM) were also prepared without the addition of EGCG.

CS/CMC/MTT-ECM scaffolds were prepared by conventional freeze-drying approach. Briefly, 200 mg CMC and 200 mg CS power were added to deionized water and stirred for 10 min. For incorporation of microspheres, 40 mg ECM was added to the mixed solution, and MMT was added subsequently. After 30 min of stirring, acetic acid (0.5% v/v) was added, and the 250 μL solution was poured into a mold. The mold was the polyfluortetraethylene plate and the area of each hole was 2 cm^2^. The mold was maintained at − 20 °C overnight, followed by lyophilization. For CS/CMC/MMT-CM and CS/CMC/MMT-EGCG scaffolds, a smilar process was conducted except 40 mg ECM was replaced by 40 mg CM and 1.2 mg EGCG, respectively.

### Characterizations

#### FT-IR analysis

The FT-IR spectrometer (Jasco-4100, JASCO, Japan) continued scanning to record the FT-IR spectra of the microspheres and scaffolds at 25 ℃ over the spectral range of 400–4000 cm^−1^ with accumulation of 16 scans and resolution of 4.0 cm^−1^.

#### XRD analysis

The microspheres and scaffolds were characterized using an analytical XPERT PRO powder diffractometer operating at a voltage of 40 kV (Cu Ka radiation) in the range of 5–75 ℃ with a 2θ step at a speed of 2θ min^−1^.

#### SEM analysis

Scanning electron microscopy (SEM, Hitachi S-3400 N, Hitachi Ltd) analysis was performed on the prepared microsphere and composite scaffolds to examine their morphology. Before observation, the samples were sputtered with gold for 50 s.

#### In vitro porosity studies

The porosity of scaffolds was evaluated as described in previous study (Yan et al. 2013). Briefly, the scaffolds were immersed in a known volume ethanol graduated cylinder (V_1_) for 5 min. Evacuation was repeated until no air bubble was discharged. The total volume was recorded as V_2_. The remaining alcohol volume after scaffolds removal was recorded as V3. The porosity of the scaffolds was calculated by the following equation:

1 $${\rm{Porosity}}{\mkern 1mu} {\mkern 1mu} \left( \% \right) = \left( {{{\rm{V}}_1}{\rm{ - }}{{\rm{V}}_3}} \right)/\left( {{{\rm{V}}_2}{\rm{ - }}{{\rm{V}}_3}} \right) \times 100.$$

#### In vitro swelling studies

The swelling ratio of the scaffolds was studied by calculating the change in the weight of the scaffolds. Dry scaffolds weight was recorded as W_d_. After immersed in distilled water for 24 h, the weight of the scaffold was recorded as W_s1_ after blotting with filter paper. Water retention ratio was evaluated by centrifuging the wet scaffolds (500 rpm, 3 min) and then recording its weight (W_s2_). The swelling and water retention ratio were obtained by the following equations:

2 $${\text{Swelling}}\,{\text{ratio}} = \left( {{\text{W}}_{{{\text{s1}}}} - {\text{W}}_{{\text{d}}} } \right)/{\text{W}}_{{\text{d}}} ,$$

3 $${\text{Retention}}\,{\text{ratio}} = \left( {{\text{W}}_{{{\text{s2}}}} - {\text{W}}_{{\text{d}}} } \right)/{\text{W}}_{{\text{d}}} .$$

#### In vitro protein adsorption studies

The equal weights scaffolds were immersed in 100% ethanol for 1 h, and then pre-wetted in 1 × phosphate buffer solution (PBS) for 30 min. The scaffold was then placed in 3 mL DMEM containing 10% fetal bovine serum and incubated at 37 °C for 1, 3 and 24 h. After the incubation, the scaffolds were removed, blotted with filter paper, and then the binding loose protein was washed off with 1 × PBS. Once the scaffold was removed, Bradford analysis was used to assess the presence of non-adsorbed proteins in the incubation solution. The amount of protein absorbed is equal to the total amount of protein minus the amount of non-absorbed protein.

#### In vitro EGCG release studies

ECM and scaffolds of equal weight were incubated at 37 °C in 1 × PBS for 30 days. The 200 μL solution were taken at predetermined time intervals and replaced with an equal volume of 1 × PBS. The absorbance of released EGCG was read at 273 nm, the concentration was calculated from an EGCG standard curve (concentrations: 20, 40, 60, 80, 100 μM). The cumulative release rate of EGCG was calculated by the following formula:

4 $${\text{Release}}\,\,\left( \% \right) = \left( {{\text{amount}}\,{\text{of}}\,{\text{EGCG}}\,{\text{released}}/{\text{initial}}\,{\text{concentration}}\,{\text{of}}\,{\text{EGCG}}\,{\text{loaded}}} \right) \times 100.$$

### Viability and proliferation of HUMSCs in the scaffolds

The HUMSCs were obtained from Ninth People’s Hospital in Shanghai. To investigate the effect of the scaffold on the activity of HUMSCs, 2 × 10^4^ cells cm^−2^ cells were seeded in the scaffold and incubated for 3 days, and then the MTT assay was performed to determine the activity of cells in the scaffold*.*

The proliferative capacity of HUMSCs in the scaffold was evaluated using the CCK-8 assay. HUMSCs were cultured in scaffolds for 1, 3, 5, and 7 days. The medium was removed, followed by the addition of 200 μL of α-MEM medium containing 10% (v/v) CCK8 solution. After incubation at 37 °C for 2 h, the OD_450_ value of the solution was determined.

In order to observe the cytoskeletal organization of HUMSCs in the scaffold, 1 × 10^4^ cells cm^−2^ of cells were seeded in the scaffold. After 3 days culture, the medium was removed. The scaffold was washed with 1 × PBS for 3 times, and then fixed in 4% (w/v) paraformaldehyde at room temperature for 15 min. The scaffold was washed again with PBS for 3 times, and then was permeated with 0.1% (w/v) Triton X-100 for 10 min. After washing the samples with PBS for 3 times, the samples were stained with phalloidin for 30 min. Samples were then washed with PBS for three times before observed with the confocal laser scanning microscope (CLSM; TCS SP8, Leica).

Cell viability was analyzed by cell death/live staining. HUMSCs (1 × 10^4^ cells cm^−2^) were inoculated in the scaffold and cultured in 24-well plates for 1, 4, and 7 days. Cells in the scaffold were washed three times using a PBS. 10 μL calcein-AM solution (2 mM) and 15 μL PI solution (1.5 mM) were added to 5 mL of a 1 × assay buffer and mixed thoroughly. The working solution concentration of calcein-AM was 4.0 μM, while that of PI was 4.5 μM. The samples were incubated with the working solution at 37 ℃ for 30 min. After washing with PBS, the samples were imaged by using a confocal laser microscope (CLSM; TCS SP8, Leica).

### In vitro osteogenic differentiation

The HUMSCs (2 × 10^4^ cells cm^−2^) seeded in different scaffolds were cultured in osteogenic induction medium for 7 days. According to the manufacturer’s instructions, the ALP activity of HUMSCs was determined qualitatively and quantitatively by BCIP/NBT alkaline phosphatase color development kit and ALP kit, respectively. Images of qualitative determination of ALP activity were captured with an Olympus MVX10 MacroView (Japan). For quantitative determination of ALP activity, HUMSCs were incubated in RIPA lysis buffer for 60 min, and the total protein concentration (mg/mL) and ALP activity (units/mL) were measured following the manufacturer’s instructions with an enhanced BCA protein assay kit and ALP reagent kit, respectively.

The HUMSCs growing on different scaffolds were cultured in osteogenic induction medium for 14 days. Calcium deposition was determined qualitatively and quantitatively by Alizarin red stain solution and calcium colorimetric assay kit according to the manufacturer’s instructions.

### Reverse transcriptase real-time (quantitative) polymerase chain reaction (RT-qPCR)

The HUMSCs (1 × 10^5^cells cm^−2^) were seeded in the scaffolds. After 14 days of culture, 5–10 × 10^5^ cells were collected in a 1.5-mL EP tube, and 500 μL TRIzol reagent was added and gently shaken to cleavage the cells, and total RNA was extracted according to the manufacturer’s instructions. The cDNA was synthesized using a kit from BioRad according to the manufacturer’s protocol, and a Quant Studio 3 real-time PCR system (Applied Biosystems, Foster City, CA, USA) and SYBR reagent were used for RT-qPCR amplification. The reaction procedure of RT-qPCR was set as follows: pre-deformation at 95 ℃ for 4 min was followed by 45 cycles (denaturation at 95 ℃ for 10 s, annealing 40 s at 60 ℃, extension at 72 ℃ for 30 s). GAPDH was used as the house keeping gene for normalization as housekeeping gene. 2^−ΔΔCt^ method was used to calculate the relative expression of genes in the HUMSCs.

### Statistical analysis

All experimental data in our study were analyzed by SPSS software, and the number of parallel experimental samples was not less than 3. Data were expressed as mean ± standard deviation (SD), *P* < 0.05 indicates significant.

## Results and discussion

### Effect of EGCG on proliferation and osteogenic differentiation of the HUMSCs

**Fig. 1:**
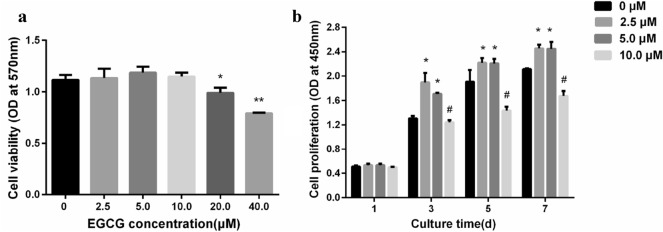
**a** Effect of EGCG on the activity of the HUMSCs after 48 h observed by MMT assay. **b** Effect of EGCG on the HUMSCs proliferation. *P < 0.05, **P < 0.01 and ***P < 0.001

Epigallocatechin-3-gallate (EGCG) is a natural flavonoid that has been shown to be involved in bone metabolism (Jin et al. 2014; Wang et al. 2016). In order to study the biocompatibility of EGCG to HUMSCs, the MTT experiment was carried out. The results in Fig. 1a show that EGCG had no significant effect on the cell activity of the HUMSCs compared to the control group at a concentration range of 2.5–10.0 μM, proving that EGCG did not produce cytotoxicity to the HUMSCs at this concentration range. However, when the concentration of EGCG was greater than 10.0 μM, the cell activity of the HUMSCs was significantly decreased by EGCG. These results indicated that 2.5–10.0 μM EGCG had the biocompatibility to the HUMSCs. In order to investigate the effect of EGCG on the proliferation of the HUMSCs, concentrations of 0, 2.5, 5.0 and 10.0 μM were set in the concentration range of 0–10.0 μM. Figure 1b shows that the number of cells increases with the extension of time. Compared with the control group, the proliferation of the HUMSCs in the experimental group supplemented with 2.5 and 5.0 μM EGCG was significantly promoted from the 3rd day of culture. In the experimental group supplemented with 10.0 μM EGCG, the proliferation of EGCG cells was significantly inhibited from the 5th day with the extension of EGCG treatment time. Therefore, 2.5–5.0 μM is the optimal concentration range for EGCG to promote cell proliferation. These results corroborated previous investigations showing the stimulatory effects of EGCG on MSCs at 2.5.0–10.0 μM in an in vitro study (Jin et al. 2014).

**Fig. 2:**
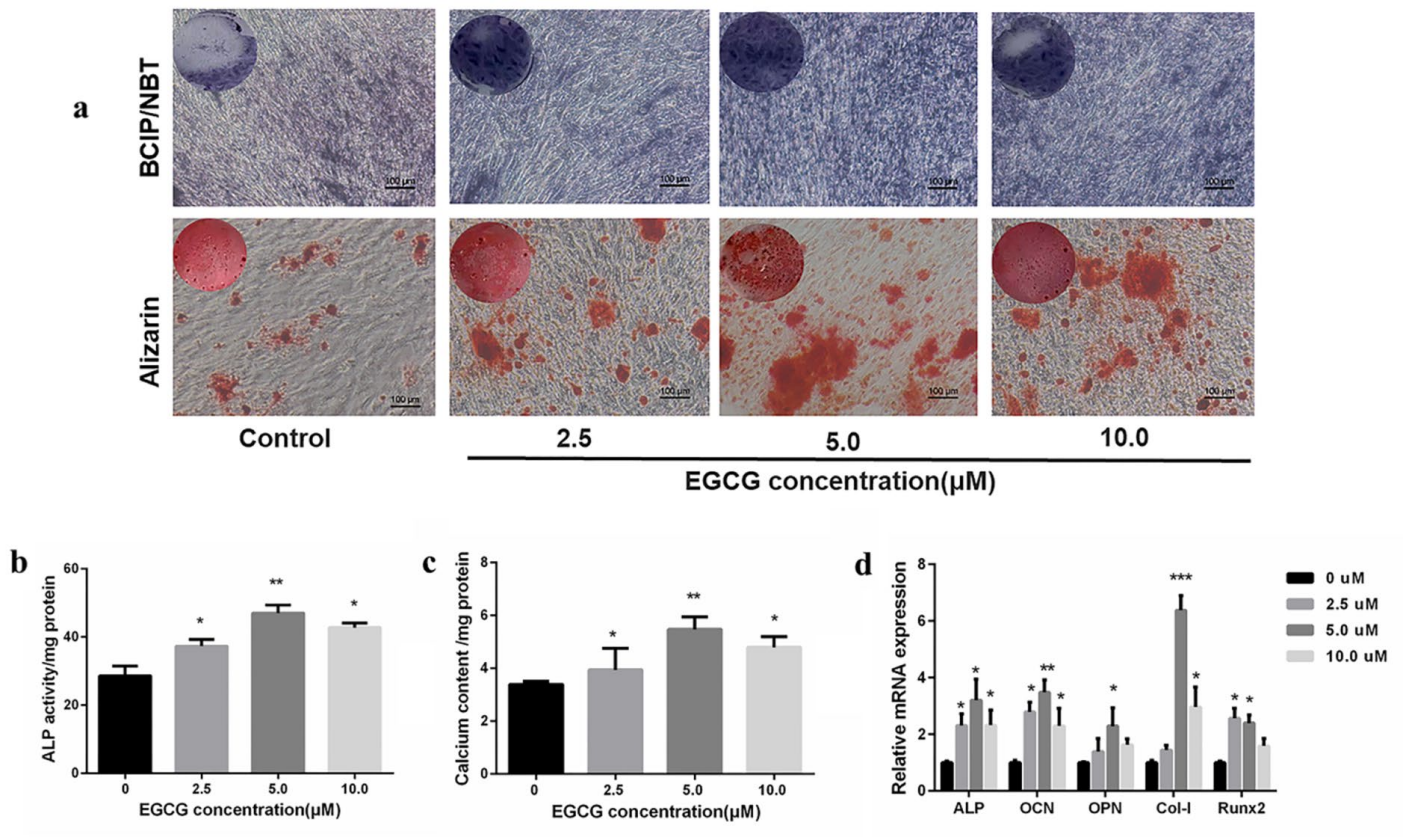
**a** The HUMSCs were cultured for 7 days for ALP staining, and the HUMSCs were cultured for 14 days for Alizarin red staining (scale bar: 100 μM). **b** Quantitative analysis of ALP activity in the HUMSCs cultured for 7 days. **c** Calcium contents in the HUMSCs cultured for 14 days. **d** The quantitative evaluation of osteogenic-related genes in the HUMSCs

In order to investigate the effect of EGCG on osteogenic differentiation of the HUMSCs and explore the optimal concentration range for promoting osteogenic differentiation, markers of osteogenic differentiation in HUMSCs at the cell level and at the molecular level were detected after a certain period of culture in osteogenic induction medium supplemented with different concentrations of EGCG. Commonly used as a marker of osteogenesis, ALP activity is assumed to reflect the degree of osteogenic differentiation (Watanabe et al. 2018). As shown in Fig. 2a, the BCIP/NBT staining results of the control group and the experimental groups with different concentrations of EGCG were blue and purple after staining, indicating the expression of ALP in all groups. However, compared with the control group, the bluish purple of all EGCG supplemental groups increased to varying degrees, indicating that ALP enzyme activity was increased in this concentration range of EGCG treatment, and the bluish purple was the most significant at 5.0 μM EGCG treatment, indicating that ALP enzyme activity was increased most obviously in the HUMSCs after 5.0 μM EGCG treatment. In addition, Alizarin red staining was used to assess the calcium content of the constructs (Zhou et al. 2014). The results of Alizarin red staining showed that there were scattered red calcium nodules in the control group. With the addition of EGCG, the number of calcium nodules increased, and the increase of calcium nodules at the concentration of 5.0 μM was the most obvious, indicating that the amount of calcium deposition at this concentration was also the largest. Subsequent quantitative determination of ALP enzyme activity and calcium deposition showed consistent results with qualitative staining (Fig. 2b, 2c).

The effects of different concentrations of EGCG on the expression of osteogenic differentiation-related genes ALP, OCN, OPN, Col-I and Runx2 were investigated, and the expression of the above five genes was determined by qRT-PCR assay. As shown in Fig. 2d, the expression of ALP, OCN and Runx2 genes were significantly upregulated by EGCG treatment at a concentration of 2.5–10.0 μM compared with the control group. However, the expression of OPN and Col-I genes were upregulated by the addition of 5.0–10.0 μM EGCG. The expression of five genes related to osteoblastic differentiation was most significantly upregulated by EGCG at 5.0 μM concentration. Taken together, these results suggested that the detection of markers of osteogenic differentiation in HUMSCs at the molecular level was consistent with that at the cellular level.

### Characterization of microspheres and scaffolds

**Fig. 3:**
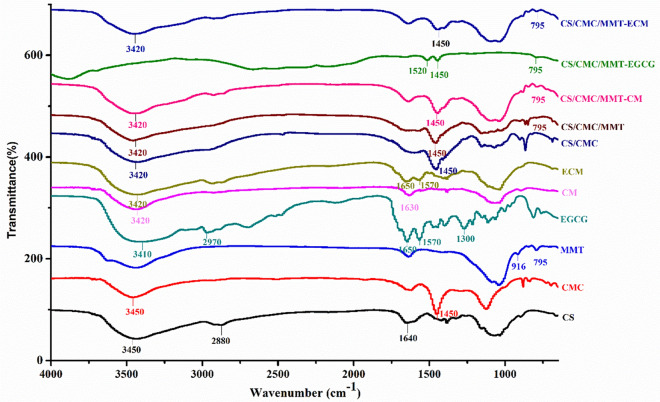
FT-IR spectra of CS, CMC, MMT, EGCG, CM, ECM, CS/CMC/MMT, CS/CMC/MMT-CM, CS/CMC/MMT-EGCG and CS/CMC/MMT-ECM scaffolds

In this study, EGCG has been proven to promote the proliferation and osteogenic differentiation of the HUMSCs in vitro. However, the EGCG is easily metabolized by cells during cell culture, which reduces its bioavailability (Li et al. 2016a, b). Therefore, ECMs were prepared and embedded in CS/CMC/MMT scaffolds to form CS/CMC/MMT-ECM scaffolds for improving the bioavailability of EGCG. FT-IR and X-ray diffraction techniques were used separately to verify whether the scaffolds were successfully formed. With reference to Fig. 3, the absorption peaks in FT-IR spectra of chitosan are at 3450 cm^−1^ (O–H and N–H, stretching), 2880 cm^−1^ (C–H, stretching) and 1640 cm^−1^ (amide II band N–H, stretching) (Koshani et al. 2021). The absorption peaks in FT-IR spectra of EGCG are at 3410 cm^−1^ (O–H, stretching), 2970 cm^−1^ (C–H, stretching), 1650 cm^−1^ (C=O, stretching), 1570 cm^−1^ (aromatic C=C, stretching), 1300 cm^−1^ (C–O, stretching). When the CM were formed, the peaks in the spectrum changed significantly. The stretching vibration of O–H and N–H at 3450 cm^−1^ in CS shifted slightly to 3420 cm^−1^. In addition, the bending vibration of N–H at 1640 cm^−1^ in CS shifted slightly to 1630 cm^−1^, indicating an augmentation of hydrogen bonding (Azizian et al. 2018; Leena et al. 2017). These results have been considered to be a connection between the aldehyde group and the amino group (Chen et al. 2017). Compared with CM, a new peak at 1650 cm^−1^ was detected in ECM. The presence of this peak indicated the successful introduction of EGCG into ECM. In addition, the characteristic peak in FT-IR spectra of CMC caused by the stretching vibration of carboxyl groups is 1710 cm^−1^ and bending vibration of C–H is 1450 cm^−1^. The characteristic stretching vibration peak in FT-IR spectra of MMT caused by Al–OH at 795 cm^−1^ were presented in the FT-IR spectra of CS/CMC/MMT. Moreover, there were characteristic peaks of CM, EGCG, ECM and CS/CMC/MMT in the FT-IR spectra of CS/CMC/MMT-CM, CS/CMC/MMT-EGCG and CS/CMC/MMT-ECM, suggesting the successful fabrication of these scaffolds.

**Fig. 4:**
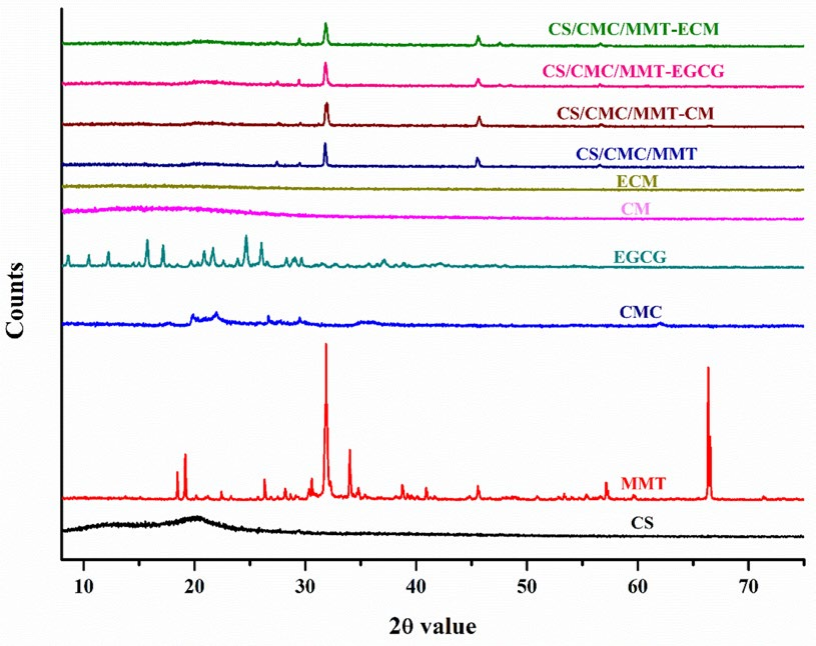
XRD patterns of CS, CMC, MMT, EGCG, CM, ECM, CS/CMC/MMT, CS/CMC/MMT-CM, CS/CMC/MMT-EGCG and CS/CMC/MMT-ECM scaffolds

The XRD pattern of CS had diffraction peaks at 2θ value of 11.2° and 20.3° as shown in Fig. 4, which shows the semi-crystal structure of CS. When CS formed CM, it will weaken the intermolecular force of chitosan and disrupt crystallization order of chitosan. Therefore, the formation of CM, the diffraction peaks observed in the XRD of the CS completely disappeared (Leena et al. 2017). The XRD pattern of EGCG showed sharp diffraction peaks at the 2θ value of 15.3° , 16.8° , 19.4° , 20.5° , 23.2° and 24.3° . However, when the ECM was formed, these diffraction peaks disappeared,indcating that EGCG was amorphized when emulsified into CM. The XRD pattern of CMC has a wide peak at the 2θ value of 20° , indicating the amorphous structure of CMC. The XRD patterns of MMT exhibited characteristic sharp peaks at the 2θ value of 19.2, 31.8, 34.0, 45.5, 57.1 and 66.4, which suggested the crystal structure of MMT. Furthermore, these characteristic peaks are also appeared in CS/CMC/MMT, but the intensity became weaker. It is possible that the addition of CS and CMC resulted in the decrease of MMT crystallinity. Compared with CS/CMC/MMT, the shape of the diffraction peaks of CS/CMC/MMT-CM, CS/CMC/MMT-EGCG and CS/CMC/MMT-ECM hardly changed, which may be due to the fact that the addition of amorphous CM, ECM and trace amount of EGCG had no effect on the crystal structure of these scaffolds.

**Fig. 5:**
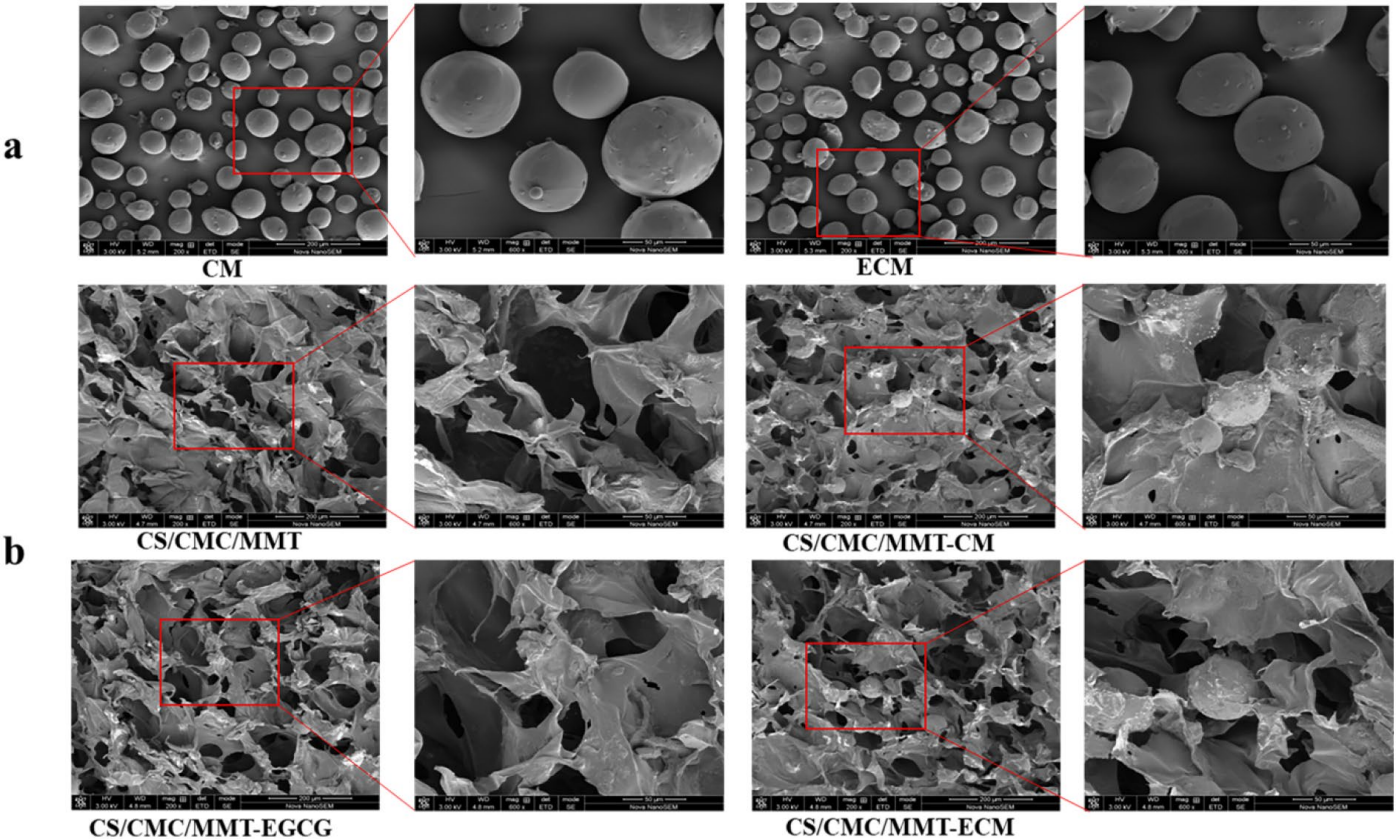
**a** SEM images of CM and ECM. **b** SEM images of CS/CMC/MMT, CS/CMC/MMT-CM, CS/CMC/MMT-EGCG and CS/CMC/MMT-ECM scaffolds. The inset shows a SEM image at 200× and 600× magnification

The morphology of microspheres and scaffolds was investigated by SEM. CM and ECM had a shape of well-defined sphere, and the average particle size of microspheres was about 40–60 μM (Fig. 5a).There was no significant difference between ECM and CM, implying that the addition of EGCG had no effect on morphological characteristics of microsphere. All scaffolds had interconnected porous structures. CM and ECM existed in CS/CMC/MMT-CM and CS/CMC/MMT-ECM scaffolds (Fig. 5b). Compared with CS/CMC/MMT scaffolds, the introduction of CM and ECM did not change the morphology of CS/CMC/MMT-CM and CS/CMC/MMT-EGCG scaffolds.

### Analysis of physicochemical properties of different scaffolds

**Fig. 6:**
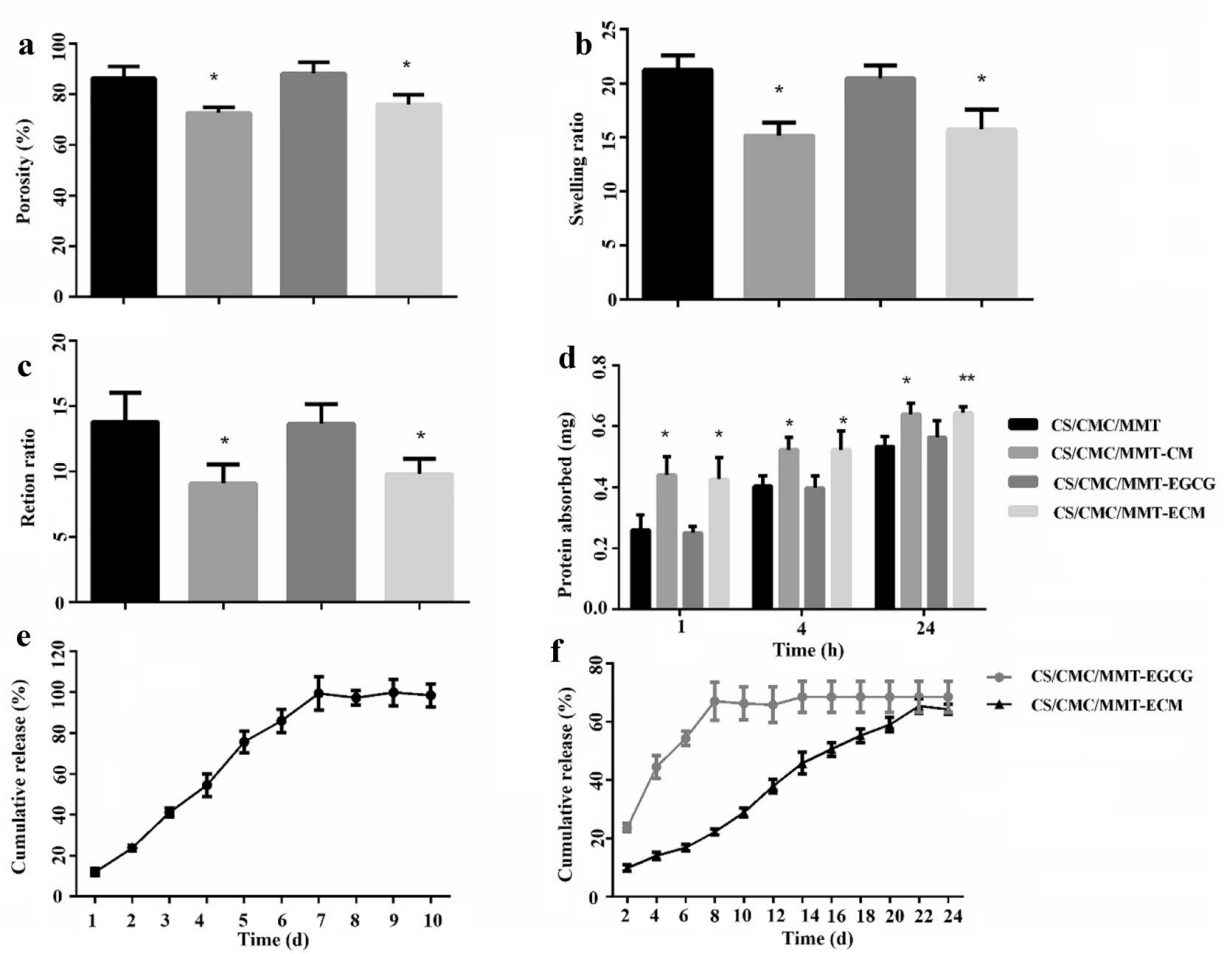
**a** The mean pore size of prepared scaffolds. **b** The water uptake of scaffolds. **c** The retention rates of scaffolds. **d** Enzymatic degradation of the scaffolds. *P < 0.05, **P < 0.01. **e** The release curve of EGCG from the ECM. **f** The release curve of EGCG from the CS/CMC/MMT-EGCG and CS/CMC/MMT-ECM scaffolds

The porosity of the scaffolds affects cell growth and nutrient permeability (Yao et al. 2018). The porosity of CS/CMC/MMT, CS/CMC/MMT-CM, CS/CMC/MMT-EGCG and CS/CMC/MMT-ECM were 72.7 ± 2.2%, 76.0 ± 3.8%, 86.3 ± 4.8% and 88.2 ± 4.6%, respectively (Fig. 6a). The porosity of CS/CMC/MMT-CM and CS/CMC/MMT-ECM scaffolds decreased significantly compared with CS/CMC/MMT and CS/CMC/MMT-EGCG scaffolds. Combined with the SEM images in Fig. 5b, it can be inferred that CM and ECM occupied the space of some open pores of the scaffolds, causing blockage of open pores, which may lead to the decrease of porosity. In addition, as shown in Fig. 6b and c, the retention ration and swelling ration of CS/CMC/MMT-CM and CS/CMC/MMT-ECM were significantly lower than those of CS/CMC/MMT and CS/CMC/MMT-EGCG.

Adsorption of proteins on the scaffolds facilitates cellular interactions, affecting cell adhesion, cell spreading and later cellular events such as proliferation and differentiation (Wilson et al. 2005). In order to evaluate the protein adsorption capacity of these scaffolds and the influence of the introduction of CM, EGCG and ECM on the scaffolds, the protein adsorption capacity of the scaffolds was tested at different times. It is noteworthy that the protein adsorption capacity of CS/CMC/MMT was basically the same as that of CS/CMC/MMT-EGCG, which proved that the direct entrapment of EGCG into CS/CMC/MMT did not affect the protein adsorption capacity of the scaffold. However, the protein adsorption capacity of CS/CMC/MMT-CM and CS/CMC/MMT-ECM was significantly higher than that of CS/CMC/MMT (Fig. 6d), implying that CM and ECM significantly enhanced the protein adsorption capacity of scaffolds.

EGCG is unstable in neutral and alkaline environments, and is easily metabolized by cells, which reduces its bioavailability (Li et al. 2016a, b). Therefore, the sustained release of EGCG is of great significance in promoting bioavailability. In order to study the sustained-release effect of EGCG from microspheres and scaffolds, the relationship between EGCG content and absorbance was determined by spectrophotometry. The standard curve equation was *Y* = 0.0127X−0.0027, *R*^2^ = 0.994. The release rate of EGCG from ECM reached 86 ± 5.6% on the 6th day, and was completely released on the 7th day (Fig. 6d). As shown in Fig. 6f, the release rate of EGCG from the CS/CMC/MMT-EGCG formed by directly encapsulated EGCG reached 67.0 ± 6.6% on the 7th day, and the release basically stopped in the subsequent time. The release rate of EGCG from CS/CMC/MMT-ECM scaffolds was 65.4 ± 2.5% on the 22nd day. The sustained-release effect of EGCG from ECM was greatly improved by filling ECM in the scaffold. In addition, compared with CS/CMC/MMT-EGCG scaffolds, CS/CMC/MMT-ECM had more significant sustained-release effect of EGCG.

### Cells proliferation and viability on different scaffolds

**Fig. 7:**
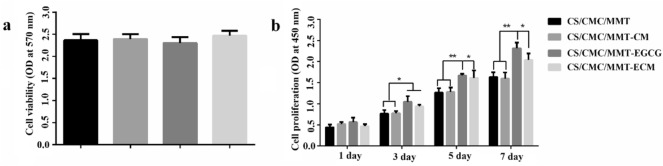
**a** The HUMSCs viability in the scaffolds after 48 h observed by MMT assay. **b** The HUMSCs proliferation in different scaffolds. *P < 0.05, **P < 0.01 and ***P < 0.001

**Fig. 8:**
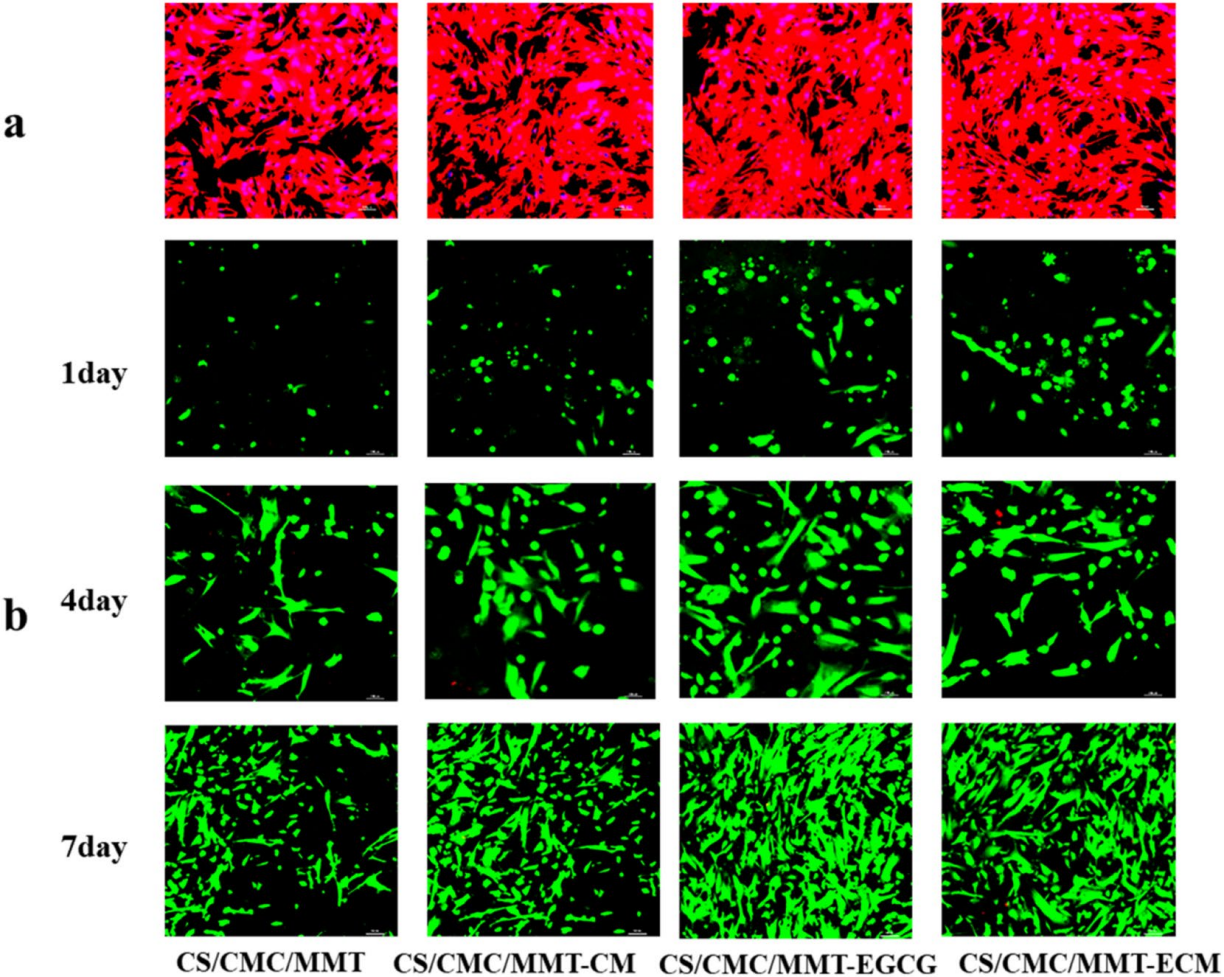
**a** Skeleton of the HUMSCs in the scaffolds; red indicates the cell's skeleton and blue indicates the nucleus (scale bar: 200 μM). **b** The live/dead staining of HUMSCs after seeding on the scaffolds for 1 days, 4 days and 7 days; green indicates the live cells and red indicates the dead cells (scale bar: 100 μM)

MTT assay was used to investigate the effect of the scaffolds on HUMSCs viability, and the results showed that the OD_570_ values of the four groups were similar (Fig. 7a), suggesting that these scaffolds was not cytotoxic. In order to further evaluate the cell viability, the HUMSCs were cultured on different scaffolds for 3 days and characterized by F-actin filament staining. Actin bundles were spindle-shaped on all four scaffolds (Fig. 8a), indicating that the cytoskeleton structure was well organized.

The proliferation of HUMSCs on these scaffolds was investigated. The results of CCK8 test showed that the OD_450_ values of the cells on these scaffolds increased continuously during the culture process (Fig. 7b). Moreover, on days 3, 5 and 7, the ability to support cell proliferation of CS/CMC/MMT-EGCG and CS/CMC/MMT-ECM scaffolds was significantly higher than that of CS/CMC/MMT-ECM and CS/CMC/MMT-CM scaffolds. In addition, as shown in Fig. 8b, the cells cultured on different scaffolds increased continuously on the 1st, 4th and 7th day of culture. There were a large number of cells on CS/CMC/MMT-EGCG and CS/CMC/MMT-ECM scaffolds, and the cells had healthy polygonal morphology. This was consistent with the results in Fig. 8b, indicating that the CS/CMC/MMT-EGCG and CS/CMC/MMT-ECM scaffolds could well maintain the activity and proliferation of HUMSCs.

### Effect of different scaffolds on osteoblast differentiation

**Fig. 9:**
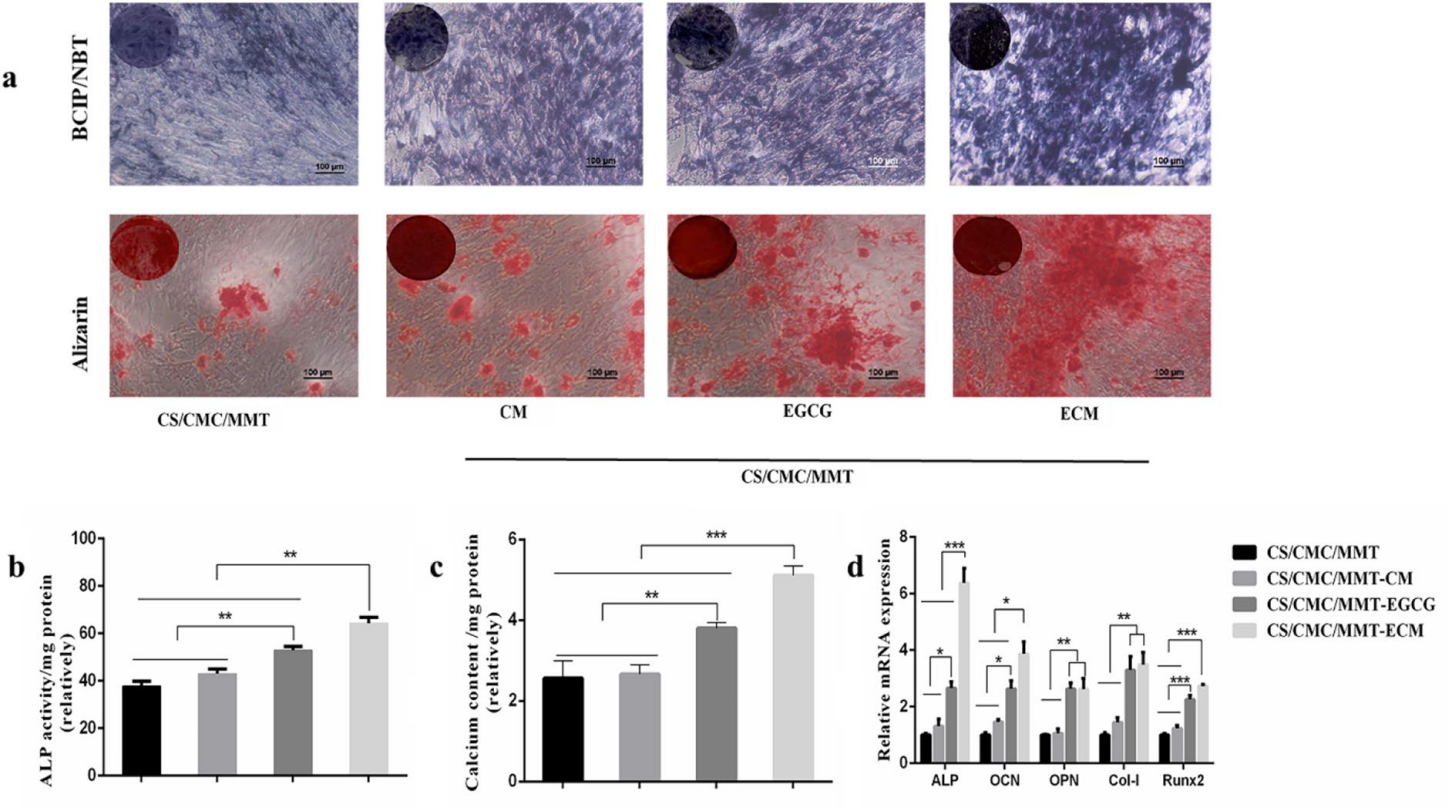
**a** The HUMSCs were cultured for 7 days for ALP staining, and the HUMSCs were cultured for 14 days for Alizarin red staining (scale bar: 100 μM). **b** Quantitative analysis of ALP activity in HUMSCs cultured for 7 days. **c** Calcium contents in HUMSCs cultured for 14 days. **d** The quantitative evaluation of osteogenic-related genes in HUMSCs

To investigate the influence of these scaffolds on osteoblast differentiation at the cellular and molecular levels, the HUMSCs seeded on the scaffold were cultured for 7 and 14 days, respectively. At the cellular level, ALP activity is assumed to reflect the degree of osteogenic differentiation (Watanabe et al. 2018). In addition, Alizarin red staining was used to assess the calcium content of the constructs (Zhou et al. 2014). As shown in Fig. 9a and Fig. 9b. Staining and quantify ALP revealed that ALP expression in CS/CMC/MMT-EGCG and CS/CMC/MMT-ECM scaffolds was enhanced. Alizarin red staining results showed that the number of calcium nodules increased significantly when HUMSCs were cultured in CS/CMC/MMT-EGCG and CS/CMC/MMT-ECM scaffolds. The quantitative calcium results (Fig. 9c) were also consistent with the qualitative staining results. Since the CS/CMC/MMT-ECM scaffolds played an important role in the promotion of HUMSCs into osteoblasts at the cellular level, we subsequently confirmed the osteogenic role of CS/CMC/MMT-ECM scaffolds at the molecular level. The osteogenic-related genes involving ALP, runt-related transcription factor 2 (Runx2), osteopontin (OPN), osteocalcin (OCN) and type I collagen (Col-I) were detected. RT-qPCR was used to investigate the expression of these genes in HUMSCs. When HUMSCs were cultured in CS/CMC/MMT-EGCG and CS/CMC/MMT-ECM scaffolds, the expression of intracellular osteogenic-related genes ALP, OCN, OPN, Co1-I and Runx2 were significantly upregulated (Fig. 9d).

## Conclusions

In this study, EGCG was entrapped in the form of ECM or monomer to form CS/CMC/MMT-ECM and CS/CMC/MMT-EGCG scaffolds, respectively. The addition of ECM decreased the porosity, swelling ration and retention ratio of the scaffolds. The results showed that the protein adsorption capacity of CS/CMC/MMT, CS/CMC/MMT-CM, CS/CMC/MMT-EGCG and CS/CMC/MMT-ECM was 0.54 ± 0.06 mg, 0.63 ± 0.05 mg, 0.56 ± 0.08 mg and 0.64 ± 0.03 mg, respectively, implying that CM and ECM significantly enhanced the protein adsorption capacity of scaffolds. In addition, compared with CS/CMC/MMT and CS/CMC/MMT-CM scaffolds, CS/CMC/MMT-EGCG and CS/CMC/MMT-ECM scaffolds had a significant promotion in the proliferation and osteoblast differentiation of the HUMSCs. Besides, CS/CMC/MMT-ECM scaffolds had a stronger effect on promoting osteogenic differentiation of the HUMSCs, which might be related to its better sustained release of EGCG.

## Data Availability

All data generated or analyzed during this study are included in this published article.

## Abbreviations

MSCs: Mesenchymal stem cells
HUMSCs: Human umbilical cord-derived mesenchymal stem cells
EGCG: Epigallocatechin-3-gallate
CS: Chitosan
CMC: Carboxymethyl cellulose
MMT: Montmorillonite
CM: CS microsphere
ECM: EGCG-encapsulated chitosan microspheres
SEM: Scanning electron microscopy
MTT: 3-(4,5-Dimethylthiazol-2yl)-2,5-diphenyltetrazolium bromide
RT-qPCR: Reverse transcriptase real-time (quantitative) polymerase chain reaction
Runx2: Runt-related transcription factor 2
OPN: Osteopontin
OCN: Osteocalcin
Col-I: Type I collagen
